# An Efficient Chemoenzymatic Approach towards the Synthesis of Rugulactone

**DOI:** 10.3390/molecules23030640

**Published:** 2018-03-12

**Authors:** Theodore Tyrikos-Ergas, Vasileios Giannopoulos, Ioulia Smonou

**Affiliations:** Department of Chemistry, University of Crete, Vasilika Vouton, 71003 Heraklion, Crete, Greece; thoriaki@yahoo.gr (T.T.-E.); billgianno@hotmail.com (V.G.)

**Keywords:** chemoenzymatic, α-pyrone, ketoreductase, bioreduction, rugulactone, inhibitor

## Abstract

Rugulactone is a natural product isolated from the plant *Cryptocarya rugulosa*. It has shown very important biological activity as an inhibitor of the nuclear factor κB (NF-κB) activation pathway. A new chemoenzymatic approach towards the synthesis of rugulactone is presented here. The chirality, induced to the key intermediate by a stereoselective enzymatic reduction utilizing NADPH-dependent ketoreductase, is described in detail.

## 1. Introduction

Rugulactone **1** is a secondary metabolite, isolated in 2009 from organic extracts of the plant *Cryptocarya rugulosa* [1]. It has shown very important biological activity and has been reported to exhibit up to five-fold induction of inhibitor of κB (IκB) proteins of nuclear factor κB (NF-κB) at 25 μg/mL [1]. The transcription factor NF-κB was discovered in 1986 as a nuclear factor regulating the formation of the κ variety of the light chain (one of two subunits from which immunoglobin is built) [2]. Soon afterwards, it became clear that these proteins, which harbor this specific DNA binding activity, are expressed in nearly all cell types. It is well known that NF-κB affects most aspects of cellular physiology—from immunity and inflammation to apoptosis, cell survival, growth, and proliferation [3,4,5,6,7]. Consistent with this role, incorrect regulation of NF-κB has been linked to cancer [8,9] and inflammatory [10,11] and autoimmune [12] diseases. Thus, there is an intensive necessity for the exploration of new biomimetic compounds that can inhibit and control NF-κB activity effectively.

**Figure 1:**
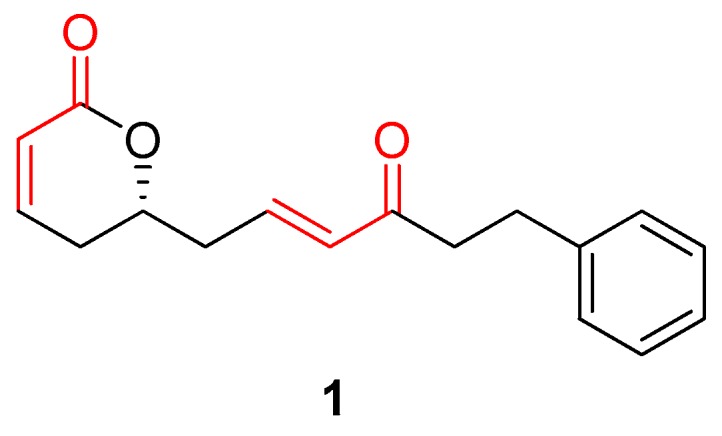
Rugulactone.

Rugulactone is a rather new dihydro α-pyrone that inhibits the NF–κB activation pathway. This natural product shows an interesting structural pattern with two electrophilic groups of potential Michael acceptors (highlighted in red, Figure 1), which covalently bind to target protein active sites, an α,β-unsaturated γ-lactone together with an α,β-unsaturated ketone. Due to the remarkable inhibitory properties of rugulactone, it has been a challenging subject of research for synthetic organic chemists. Recent studies have verified that many analogues [13,14] and derivatives [8] of rugulactone demonstrate similar or better antibacterial and antifungal activity as compared to the natural product, even with the opposite configuration of the natural product. Several synthetic schemes have been reported where chirality has been induced using various methods [13,14,15,16,17,18,19,20,21,22], such as a chiral pool [20], proline-catalyzed α-aminooxylation of aldehydes [13], Jacobsen’s hydrolytic kinetic resolution of epoxides [16], Keck’s asymmetric allylation [17], allylation of carbonyl compounds with chiral boronic esters [19], or asymmetric catalytic Overman esterification [14]. Also, a chemoenzymatic synthesis was reported by Fadnavis et al. [18], involving an enzymatic kinetic resolution of racemic homoallylic alcohols employing *Candida rugosa* lipase. However, in this case, the maximum conversion to the desired enantiomer could not exceed 50%. The majority of all of these synthetic routes include several synthetic steps and utilize expensive starting materials and peculiar reagents.

Biocatalysis has long been known as an alternative technology, capable of delivering highly stereo-, chemo-, and regioselective transformations [23,24]. Recent contributions demonstrate the broad diversity of impressive opportunities for chemoenzymatic processes, which underline their potential as valuable solutions for current synthetic challenges in the synthesis of valuable chiral building blocks towards the production of pharmaceuticals and biologically active natural products [23,24,25,26].

As a part of our interest in applications of bioreductions in the synthesis of biologically interesting compounds, we report here a new chemoenzymatic approach towards the synthesis of rugulactone **1**.

## 2. Results and Discussion

**Scheme 1:**
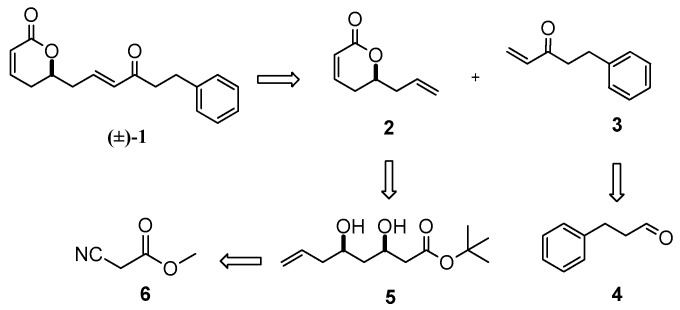
Retrosynthetic analysis of rugulactone.

Our synthetic approach consisted of eight simple synthetic steps utilizing inexpensive starting materials. The chirality of the natural product was achieved by a stereoselective enzymatic reduction utilizing NADPH-dependent ketoreductase. Initially, the racemic rugulactone was synthesized according to the retrosynthetic approach shown in Scheme 1.

Rugulactone (±)-**1** can be derived from a Grubbs’ cross metathesis between the two terminal alkenes **2** and **3**. Lactone **2** can be synthesized from the dihydroxy ester **5**, which can be derived from the commercially available methyl-2-cyanoacetate **6**. On the other hand, enone **3** can be obtained easily from the commercially available aldehyde **4**.

**Scheme 2:**
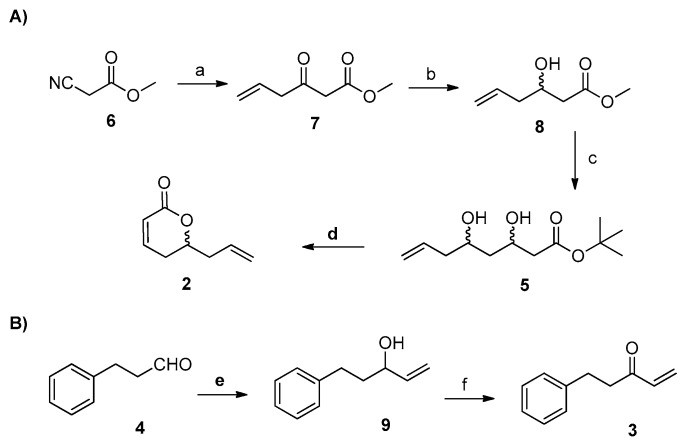
(**A**) Reagents and conditions: (**a**) allyl bromide, Zn powder, tetrahydrofuran (THF), 0 °C–r.t., 52%; (**b**) NaBH_4_, MeOH, 0 °C, 95%; (**c**) (i) lithium diisopropylamide (LDA), *tert*-butyl acetate, THF, −78 °C (ii) NaBH_4_, MeOH, 0 °C, 79% over two steps; (**d**) *p*-toluenesulfonic acid (PTSA), toluene, reflux, 97%; (**B**) (**e**) vinyl magnesium chloride, THF, 0 °C–r.t., 86%; (**f**) 2-iodoxybenzoic acid (IBX), dimethyl sulfoxide (DMSO), r.t., 88%.

Initially, the synthesis of intermediate **5** was achieved through keto ester **7** (Scheme 2). The synthesis of compound **7** was reported many years ago [27,28]. Unfortunately, in our case, the classic C–C coupling involving Claisen or Grignard, as well as Reformatsky and aldol, reactions led to unexpected byproducts. Therefore, these synthetic methods were not useful. Next, we chose an alternative and simpler approach for the synthesis of **7**, utilizing an organometallic Barbier reaction [29]. The carbonyl moiety was then reduced by NaBH_4_ in MeOH to furnish the corresponding hydroxy ester **8**. In addition, compound **5** was obtained from a Claisen reaction between the hydroxy ester **8** and commercially available *tert*-butyl acetate, followed by a carbonyl reduction. Furthermore, a single-step cyclization of dihydroxy compound **5** with simultaneous dehydration, catalyzed by *p*-toluenesulfonic acid, led to γ-lactone **2** in good yield (Scheme 2).

**Scheme 3:**
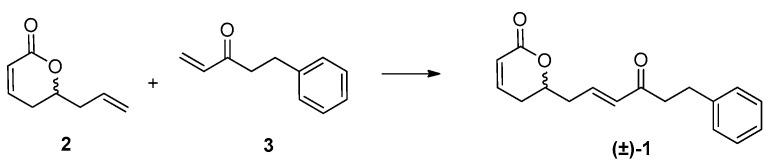
Reagents and conditions: Grubbs(II) catalyst (5 mol %), dry CH_2_Cl_2_, 45 °C, 75%.

Furthermore, enone **3** was synthesized from commercially available 3-phenylpropanal **4** in two steps involving alkylation of the aldehyde through Grignard vinylation followed by 2-iodoxybenzoic acid (IBX) oxidation of the resulting allylic alcohol **9** (Scheme 2). Finally, the cross-metathesis of the two terminal alkenes—the lactone **2** and the enone **3**—catalyzed by Grubbs (II) catalyst [30], led successfully to the formation of (±)-rugulactone **1** in a good reaction yield of 75%, whereas the overall yield of the synthesis was found to be 21% (Scheme 3). The spectroscopic data are identical to those reported in the literature [1].

**Scheme 4:**
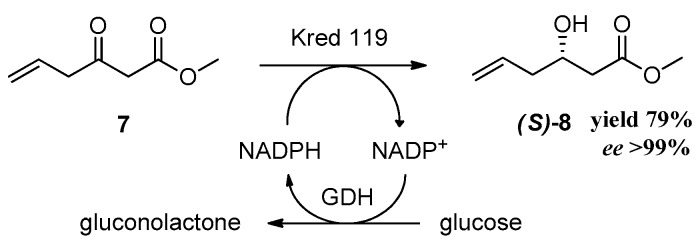
Asymmetric enzymatic reduction of **7** using Kred 119.

After the successful completion of this attractive synthetic procedure of racemic rugulactone, we applied this methodology for the synthesis of the optically active rugulactone **1** by the intervention of a key enzymatic step. Our research group has lengthy experience in biocatalytic reductions of various carbonyl compounds and their applications for the chemoenzymatic synthesis of natural products with biological activity [31,32]. Moreover, we and other research groups have shown that reductive enzymes, like ketoreductases, are valuable and powerful catalysts in the synthesis of optically active intermediates and precursors for many pharmaceuticals [33,34,35,36,37,38,39,40]. In the present work, the asymmetric reduction of the keto ester **7** using NADPH-dependent ketoreductases was the key step introducing the chirality of the natural product **1**. Before conducting the screening for active enzymes, we tested the stability of **7** under the usual enzymatic reaction conditions (phosphate buffer 200 mM, pH 6.9, and 37 °C) [33,34]. Unfortunately, under those conditions, substrate **7** was decomposed, as it was observed by the ^1^H-NMR spectroscopy. After the clarification of the optimum conditions (phosphate buffer 200 mM, pH 6.9, and temperature 5–8 °C), the stereoselective reduction of **7** was carried out with several ketoreductases to screen and identify the most suitable biocatalyst for the desirable transformation. The enzymes chosen for the screening have displayed high activity in structurally similar carbonyl substrates [32,36,37]. The well-established system for the NADPH recycling was applied in all enzymatic reactions (Scheme 4) [33]. Many enzymes demonstrated activity towards the reduction of **7**, and the best results are shown in Table 1.

Ketoreductases (Kreds) A1C, A1D, B1F, 101, and 119 showed excellent activity (>99% conv.) towards the reduction of keto ester **7** (conv > 99%). Kreds A1C, A1D, and 119 displayed excellent enantioselectivity (*ee* > 99%) (Scheme 4), whereas Kreds B1F and 101 showed reasonable enantioselectivity. All positive enzymes catalyzed efficiently the reduction to form the optically active hydroxy ester **8**. The absolute configuration of the hydroxy ester was determined by assigning the stereochemistry of the hydroxy group by the use of methoxy phenyl acetic esters (MPA esters) [41] and was found to be the (*S*)-**8** enantiomer. Compound **8** is the key chiral synthon for the chemoenzymatic synthesis of the (*S*)-epimer of natural product **1** according to our proposed synthesis (Scheme 2). The synthesis of this epimer is very useful since it has demonstrated high biological activity like many analogues [15] and derivatives [8].

**Scheme 5:**
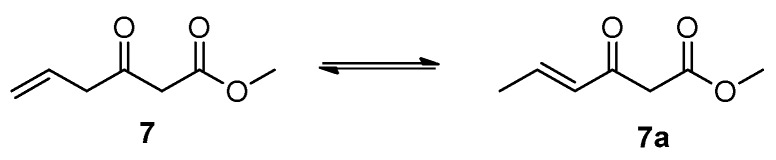
Isomerization of keto ester **7**.

It is worth noting that under the enzymatic reaction conditions, substrate **7** showed a double bond isomerization to form a small amount (6%) of compound **7a**, as it was observed by ^1^H-NMR spectroscopy (Scheme 5).

For this reason, the final product of the enzymatic reduction in every case was a mixture of the desired optically active hydroxy ester **8** and a side product derived from the double bond reduction as well as the carbonyl moiety reduction of the isomer **7a**. This observation was in accordance with our previous unpublished results with several ketoreductases, which can also reduce α,β-unsaturated keto moieties together with the double bond reduction. This can most likely be attributed to contaminations of these commercially available enzymes.

## 3. Materials and Methods

### 3.1. General

Unless otherwise noted, all solvents and reagents were purchased from Sigma-Aldrich (Munich, Germany) in the highest purity and were used without any further purification. Dry tetrahydrofuran (THF) was used after distillation into a Soxhlet in the presence of metallic Na and benzophenone. Kreds, glucose dehydrogenase, and NADPH were commercially available (Codexis, Redwood City, CA, USA). NMR spectra were generally recorded at room temperature on Bruker Avance series 500 and 300 spectrometers (Billerica, MA, USA). Chemical shifts (*δ*) are reported in ppm relative to the residual solvent peak (CDCl_3_, *δ*: 7.26, ^13^CDCl_3_, *δ*: 77.0), and the multiplicity of each signal is designated by the following abbreviations: s, singlet; d, doublet; t, triplet; q, quartet; m, multiplet; br, broad. Coupling constants (*J*) are quoted in Hz. ^1^H- and ^13^C-NMR spectra of compounds **1**–**3**, **5**, and **7**–**9**, and ^1^H-NMR of (*R*)-MPA and (*S*)-MPA esters of (*S*)-methyl 3-hydroxyhex-5-enoate can be found in Supplementary Materials. Thermo LTQ-Orbitap XL with an electron transfer dissociation (ETD) ion trap mass spectrometer (Waltham, MA, USA) was used for the high resolution mass spectra (HRMS). Column chromatographic separations were carried out of a flash chromatography system using silica gel and hexane/ethyl acetate or petroleum ether/ethyl acetate solvent mixtures. For thin layer chromatography (TLC), Merck silica gel (grade 60 F_245_, Merck & Co., Kenilworth, NJ, USA) was used. The progress of the enzymatic reactions and the selectivities were determined by gas chromatography using SHIMADZU GC-2014 gas chromatograph (Shimadzu, Japan) equipped with an flame ionization detector (FID) detector (HP GC column: 30 m × 0.25 mm × 0.25 μm chiral capillary column, 20% permethylated, Cyclodextrin-B, Part No. 19091G-B233).

### 3.2. Synthetic Procedures

#### 3.2.1. Methyl 3-Oxohex-5-enoate (**7**)

Aluminum trichloride (160 mg, 1.2 mmol) was added all at once to a solution of zinc powder (780 mg, 12 mmol), methyl 2-cyanoacetate (267 μL, 3 mmol), and crotyl bromide (390 μL, 4.5 mmol) in anhydrous THF (12 mL) at 0 °C (ice-water bath). The reaction mixture was warmed to room temperature and then stirred at room temperature. After the reaction was completed (monitored by TLC), aqueous HCl (2 M, 5 mL) was added to the reaction mixture and was stirred at room temperature for 5 min. The reaction mixture was passed through a short silica gel column and the organic solvent was removed directly under reduced pressure. Further purification was achieved by flash column chromatography (hexane/EtOAc, *v*/*v*, 30/1) to give the corresponding ester **7** with 52% isolated yield (222 mg). ^1^H-NMR (500 MHz; CDCl_3_; Me_4_Si): *δ* 5.87–5.95 (m, 1H), 5.22–5.25 (m,1H), 5.16–5.20 (m, 1H), 3.74 (s, 3H), 3.50 (s, 2H), 3.305 (d, *J* = 7.0 Hz, 2H). ^13^C-NMR (500 MHz, CDCl_3_): *δ* 200.5, 167.45, 129.5, 119.8, 52.4, 48.3, 47.7.

#### 3.2.2. Methyl 3-Hydroxyhex-5-enoate (**8**)

Sodium borohydride (0.26 mmol, 10 mg) was added in dry ethanol (10 mL) under nitrogen, and the mixture was cooled to 0 °C. After stirring for 5 min a solution of dry methanol (5 mL) containing methyl 3-oxohex-5-enoate **7** (111 mg, 0.78 mmol) was added dropwise. After stirring for 4 h at 0 °C, the reaction was quenched with saturated ammonium chloride, and methanol was concentrated by rotor evaporator. Then, water (20 mL) was added and extracted twice with EtOAc (2 × 20 mL). The organic layer was dried over MgSO_4_ and the solvents were evaporated to dryness. The residue was purified by flash column chromatography (hexane/EtOAc, *v*/*v*, 20/1) to afford the methyl 3-hydroxyhex-5-enoate **8** with 95% yield (107 mg). ^1^H-NMR (500 MHz; CDCl_3_; Me_4_Si; δ ppm): *δ* 5.78–5.86 (m, 1H), 5.13–5.16 (m, 1H), 5.11–5.12 (m, 1H), 4.06–4.12 (m, 1H), 3.71 (s, 3H), 2.53 (dd, *J*_1_ = 16.6 Hz, *J*_2_ = 3.5 Hz, 1H), 2.44 (dd, *J*_1_ = 16.6 Hz, *J*_2_ = 9 Hz, 1H), 2.23–2.33 (m, 2H). ^13^C-NMR (500 MHz, CDCl_3_): *δ* 173.2, 133.9, 118.2, 67.3, 51.8, 40.9, 40.4.

#### 3.2.3. *tert*-Butyl 3,5-dihydroxyoct-7-enoate (**5**)

Under nitrogen atmosphere, dry diisopropyl amine (621 μL, 4.72 mmol) was dissolved in dry THF (5 mL). The mixture was cooled to 0 °C and BuLi 1.6 M in hexane (3.0 mL, 4.4 mmol) was added dropwise. After stirring for 20 min at 0 °C the mixture was cooled to −78 °C and a solution of *tert*-butyl acetate (439 mg, 2 mmol) in dry THF (2 mL) was added and the mixture was stirred at −78 °C for 20 min. Then, the mixture was cooled to −78 °C and a solution of methyl 3-hydroxyhex-5-enoate **8** (340 mg, 2.36 mmol) in dry THF (3 mL) was added and the reaction mixture was stirred until the completion of the reaction, which was observed by TLC analysis of reaction aliquots. The reaction mixture was quenched with saturated NH_4_Cl (20 mL) and was extracted with EtOAc (2 × 15 mL). The combined organic layers were dried over MgSO_4_ and evaporated to dryness. Then, the crude product was dissolved in dry methanol (5 mL) and the methanol mixture was added dropwise to a stirring solution of sodium borohydride (45 mg, 0.87 mmol) in dry methanol (8 mL) under nitrogen at 0 °C. The reaction mixture was stirred until the completion of the reaction monitored by TLC. The reaction was quenched with saturated ammonium chloride, and the methanol was concentrated by rotor evaporator. Then, water (20 mL) was added and the aqueous layer was extracted twice with EtOAc (2 × 20 mL). The organic layer was dried over MgSO_4_ and the solvents were evaporated to dryness. The residue was purified using silica gel chromatography (hexane/EtOAc, *v*/*v*, 15/1) to afford the *tert*-butyl 3,5-dihydroxyoct-7-enoate **5** with 79% overall yield (363 mg). ^1^H-NMR (500 MHz; CDCl_3_; Me_4_Si): *δ* 5.78–5.86 (m, 1H), 5.07–5.14 (m, 2H), 4.20–4.42 (m, 1H), 3.90–4.15 (m, 1H), 2.36–2.63 (m, 2H), 2.20–2.36 (m, 2H), 1.52–1.70 (m, 2H), 1.46 (s, 9H). ^13^C-NMR (500 MHz, CDCl_3_): *δ* 172.4, 172.08, 134.6, 134.5, 118, 117.8, 81.5, 71.1, 69.0, 67.5, 65.7, 43.2, 42.5, 42.1, 42.0, 41.5, 41.4, 28.1. HRMS (TOF-ESI) *m*/*z*: [M + H]^+^ Calcd. for C_12_H_22_O_4_+H, 231.1552; Found 231.1592.

#### 3.2.4. Allyl-5,6-dihydro-2*H*-pyran-2-one (**2**)

Under nitrogen atmosphere, *tert*-butyl 3,5-dihydroxyoct-7-enoate **5** (100 mg, 0.46 mmol) was dissolved in dry toluene (8 mL) and *p*-toluenesulfonic acid monohydrate (45 mg, 0.26 mmol) was added. After stirring the solution at room temperature for 5 min, the reaction mixture was refluxed for 5 h. After completion of the reaction, the reaction mixture was washed twice with sodium carbonate (2 × 8 mL). The organic layer was dried over MgSO_4_, and the solvent was evaporated to dryness. Pure 6-allyl-5,6-dihydro-2*H*-pyran-2-one **2** was obtained after silica gel chromatography (hexane/EtOAc *v*/*v*, 10/1) with 97% yield (62 mg). ^1^H-NMR (500 MHz; CDCl_3_; Me_4_Si; *δ* ppm): *δ* 6.86–6.90 (m, 1H), 6.02 (d, *J* = 9.7 Hz, 1H), 5.79–5.88 (m, 1H), 5.15–5.19 (m, 2H), 4.46–4.51 (m, 1H), 2.53–2.59 (m, 1H). 2.44–2.50 (m, 1H), 2.33–2.36 (m, 2H) ^13^C-NMR (500 MHz, CDCl_3_): *δ* 164.3, 145.0, 132.2, 121.4, 118.9, 77.0, 39.0, 28.6.

#### 3.2.5. 5-Phenylpent-1-en-3-ol (**9**)

To an ice-cold solution of 3-phenylpropanal **8** (700 μL, 5.14 mmol) and dry THF (8 mL), vinyl magnesium chloride 1 M (1.5 mL, 1.4 mmol) was added dropwise and the reaction was stirred at room temperature for 3 h under nitrogen dry atmosphere. When the reaction was complete (TLC), it was quenched with saturated aqueous NH_4_Cl, and the mixture was extracted with Et_2_O (3 × 50 mL). The combined organic layers were dried (MgSO_4_) and concentrated under reduced pressure. The residue was purified by column chromatography (hexane/EtOAc *v*/*v*, 20/1) to produce the corresponding homoallylic alcohol as a clear liquid with 86% isolated yield (195 mg). ^1^H-NMR (500 MHz; CDCl_3_; Me_4_Si): *δ* 7.27–7.30 (m, 2H), 7.17–7.216 (m, 3H), 5.9 (ddd, *J*_1_ *=* 17.2 Hz, *J*_2_ = 10.4 Hz, *J*_3_ = 6.2 Hz, 1H), 5.23–5.27 (m, 1H), 5.12–5.15 (m, 1H), 4.11–4.14 (m, 1H), 2.66–2.78 (m, 2H), 1.80–1.91 (m, 2H). ^13^C-NMR (300 MHz, CDCl_3_): *δ* 141.8, 141.0, 128.5, 128.40, 125.9, 115.0, 72.5, 38.5, 31.6.

#### 3.2.6. 5-Phenylpent-1-en-3-one (**3**)

The homoallylic alcohol **9** obtained above (0.5 mL, 4.92 mmol) was added to a stirred solution of IBX (1.4 g, 5.14 mmol) in dry DMSO (10 mL) at room temperature, and the mixture was stirred for 6 h. When the reaction was complete, the mixture was filtered, diluted with H_2_O (10 mL), and extracted with CH_2_Cl_2_ (2 × 20 mL). The combined organic layers were washed with brine (20 mL), dried (MgSO_4_), and concentrated in vacuo. Purification by column chromatography (petroleum ether/EtOAc *v*/*v*, 25/1) furnished compound **3** with 88% isolated yield (693 mg). ^1^H-NMR (500 MHz; CDCl_3_; Me_4_Si): *δ* 7.28–7.30 (m, 2H), 7.19–7.21 (m, 3H), 6.36 (dd, *J*_1_ = 17.8 Hz, *J*_2_ = 10.6 Hz, 1H)), 6.22 (d, *J* = 17.8 Hz, 1H), 5.83 (d, *J* = 10.6 Hz, 1H), 2.90–2.98 (m, 4H). ^13^C-NMR (500MHz, CDCl_3_): *δ* 199.8, 141.0, 136.5, 128.5, 128.33, 128.27, 126.1, 41.2, 29.8.

#### 3.2.7. (*E*)-6-(4-Oxo-6-phenylhex-2-enyl)-5,6-dihydro-2*H*-pyran-2-one (**1**)

Under nitrogen atmosphere, Grubbs’ (II) catalyst (15 mg, 5 mol %) was added to a stirred solution of 6-allyl-5,6-dihydro-2*H*-pyran-2-one **2** (50 mg, 0.36 mmol) and 5-phenylpent-1-en-3-one **3** (174 mg, 1.1 mmol) in dry CH_2_Cl_2_ (10 mL), and the reaction mixture was stirred at 45 °C for 12 h. After completion of the reaction, the solvent was evaporated to dryness and the residue was purified using silica gel chromatography (hexane/EtOAc, *v*/*v*, 4/1) to afford **1** with 75% isolated yield (73 mg). ^1^H-NMR (500 MHz; CDCl_3_; Me_4_Si): *δ* 7.26–7.29 (m, 2H), 7.17–7.20 (m, 3H), 6.86–6.90 (m, 1H), 6.77–6.83 (m, 1H), 6.19 (d, *J* = 15.9 Hz), 6.04 (dt, *J*_1_ = 9.8 Hz, *J*_2_ = 1.8 Hz, 1H), 4.52–4.57 (m, 1H),2.88–2.96 (m, 4H), 2.58–2.70 (m, 2H), 2.32–2.34 (m, 2H). ^13^C-NMR (500 MHz, CDCl_3_): *δ* 199.0, 163.7, 144.6, 141.0, 140.0, 133.5, 128.5, 128.4, 126.1, 121.5, 76.0, 41.7, 37.5, 29.9, 28.9.

### 3.3. General Procedure for Enzymatic Reductions

The reductions were performed as follows: In a phosphate buffered solution (1 mL, 200 mM, pH 6.9), the substrate (5 mg, 0.035 mmol), the corresponding Kred (2 mg), glucose (21 mg), glucose dehydrogenase (2 mg), and NADPH (2 mg, 2.5 mM) were added. The reactions were incubated at 3–8 °C. After completion of the reactions, the products were isolated by extracting the crude reaction mixture with EtOAc (3 × 1.5 mL). The combined organic layers were dried over MgSO_4_ and evaporated to dryness.

#### (*S*)-Methyl 3-hydroxyhex-5-enoate, (*S*)-**8**

The enzymatic reduction of methyl 3-oxohex-5-enoate **7** catalyzed by Kred-119 was completed after 3 h and the optically active alcohol **8** was produced with 79% yield and > 99% *ee*. The spectroscopic data are identical to those of compound **8.** ^1^H-NMR (500 MHz; CDCl_3_; Me_4_Si): *δ* 5.78–5.86 (m, 1H), 5.13–5.16 (m, 1H), 5.11–5.12 (m, 1H), 4.06–4.12 (m, 1H), 3.71 (s, 3H), 2.53 (dd, *J*_1_ = 16.6 Hz, *J*_2_ = 3.5 Hz, 1H), 2.44 (dd, *J*_1_ = 16.6 Hz, *J*_2_ = 9 Hz, 1H), 2.23–2.33 (m, 2H). ^13^C-NMR (500 MHz, CDCl_3_): *δ* 173.2, 133.9, 118.2, 67.3, 51.8, 40.9, 40.4. GC data: (column: 30 m × 0.25 mm × 0.25 μm chiral capillary column, Cyclodextrin-B 150 °C for 10 min, rate: 0 °C/min; carrier gas: He, press 90 kPa). *t*_R_ = 2.61 min.

### 3.4. Preparation of MPA-Esters

#### 3.4.1. General Method for the Synthesis of MPA Esters of Secondary Alcohols

To a solution of the corresponding secondary alcohol (0.1 mmol) in dry CH_2_Cl_2_ were added 1.1 equivalent of *N*,*N*’-dicyclohexylcarbodiimide (DCC) (0.11 mmol, 23 mg) and 1.1 equivalent of the corresponding (*R*) or (*S*) MPA (0.11 mmol, 18 mg) and the reaction mixture was stirred at 0 °C for 4–6 h. After completion of the reaction, the produced urea was filtered and the filtrate was evaporated and then purified by column chromatography with 5/1 Hex/EtOAc. The produced corresponding MPA-ester was isolated with 90% isolated yield.

##### (*R*)-MPA Ester of (*S*)-Methyl 3-hydroxyhex-5-enoate

^1^H NMR (500 MHz; CDCl_3_; Me_4_Si): *δ* 7.40–7.42 (m, 2H), 7.317–7.36 (m, 3H), 5.41–5.49 (m, 1H), 5.30–5.35 (m, 1H), 4.84–4.90 (m, 2H), 4.72 (s, 1H), 3.60 (s, 3H), 3.41 (s, 3H), 2.52–2.62 (m, 2H), 2.218–2.223 (m, 2H).

##### (*S*)-MPA Ester of (*S*)-Methyl 3-hydroxyhex-5-enoate

^1^H NMR (500 MHz; CDCl_3_; Me_4_Si): *δ* 7.40–7.42 (m, 2H), 7.31–7.36 (m, 3H), 5.65–5.74 (m, 1H), 5.26–5.35 (m, 1H), 5.05–5.08 (m, 2H), 4.71 (s, 1H), 3.39 (s, 3H), 3.37 (s, 3H), 2.44–2.50 (m, 2H), 2.37–2.41 (m, 2H).

## 4. Conclusions

In summary, a total synthesis of (±)-rugulactone has been achieved in a highly efficient and concise way—in eight steps with an overall yield of 21%. This flexible synthetic pathway can be applied to the synthesis of a variety of α,β–unsaturated α-pyrones, which bear two potential Michael acceptors. Concerning the chemoenzymatic synthetic approach of the optically active (S) epimer of the natural product 1, we identified through a screening procedure three suitable highly stereoselective ketoreductases, which catalyzed efficiently the formation of the chiral key intermediate **8** as the (*S*) enantiomer in high yield and excellent *ee*, using ketoreductases for the first time. Further studies focused on screening other anti-Prelog reductive enzymes for the synthesis of the (*R*)-**8** enantiomer are currently underway.

## Figures and Tables

**Table 1:** Enzymatic reduction of methyl 3-oxohex-5-enoate **7**.

Substrate	Kred ^a^	Conv. ^b^ (time)		Yield ^c^	*ee* ^d^	Product
7	A1C	>99%	(12 h)	62%	>99%	(*S)*-**8**
	A1D	>99%	(12 h)	64%	98%	(*S)*-**8**
	119	>99%	(12 h)	79%	>99%	(*S)*-**8**
	B1F	>99%	(12 h)	62%	58.6%	(*S)*-**8**
	101	>99%	(12 h)	83%	72%	(*S)*-**8**

^a^ Ketoreductase; ^b^ Conversions were derived from ^1^H-NMR spectra; ^c^ Isolated; ^d^ Enantiomeric excess was derived from chiral column GC analysis.

## Supplementary Materials

Supplementary materials are available online at http://www.mdpi.com/1420-3049/23/3/640/s1. ^1^H- and ^13^C-NMR of compound **7**: S2, ^1^H- and ^13^C-NMR compound **8**: S3, ^1^H- and ^13^C-NMR compound **5**: S4, ^1^H- and ^13^C-NMR compound **2**: S5, ^1^H- and ^13^C-NMR compound **9**: S6, ^1^H- and ^13^C-NMR compound **3**: S7, ^1^H- and ^13^C-NMR compound **1**: S8, ^1^H-NMR of (*R*)-MPA and (*S*)-MPA esters of (*S*)-methyl 3-hydroxyhex-5-enoate: S9.
