# Supplementary material for: An Efficient Chemoenzymatic Approach towards the Synthesis of Rugulactone
Source: Molecules. 2018 Mar 12;23(3):640. doi: 10.3390/molecules23030640 (PMC6017073; doi:10.3390/molecules23030640)

SUPPLEMENTARY MATERIAL FOR

An efficient chemoenzymatic approach towards the synthesis of Rugulactone

Theodore Tyrikos-Ergas[a], Vasileios Giannopoulos[a], Ioulia Smonou[a]*
[a] *Department of Chemistry, University of Crete, Vasilika Vouton, 71003 Heraklion, Crete, Greece*

Table of Contents:

- Title, Affiliations, Table of Contents....................S1

1. Copies ^{1}H and ^{13}C NMR of compounds **1-3, 5, 7-9**....................S2-S8
   1.1. Copies ^{1}H and ^{13}C NMR compound **7**....................S2
   1.2. Copies ^{1}H and ^{13}C NMR compound **8**....................S3
   1.3. Copies ^{1}H and ^{13}C NMR compound **5**....................S4
   1.4. Copies ^{1}H and ^{13}C NMR compound **2**....................S5
   1.5. Copies ^{1}H and ^{13}C NMR compound **9**....................S6
   1.6. Copies 1H and 13C NMR compound **3**....................S7
   1.7. Copies 1H and 13C NMR compound **1**....................S8

2. Copies ^{1}H NMR of (*R*)-MPA and (*S*)-MPA esters of (*S*)-methyl 3-hydroxyhex-5-enoate.........S9

1. Copies ^{1}H and ^{13}C NMR of compounds 1-3, 5, 7-9

1.1 *Methyl 3-oxohex-5-enoate* **7** ($CDCl_3$)

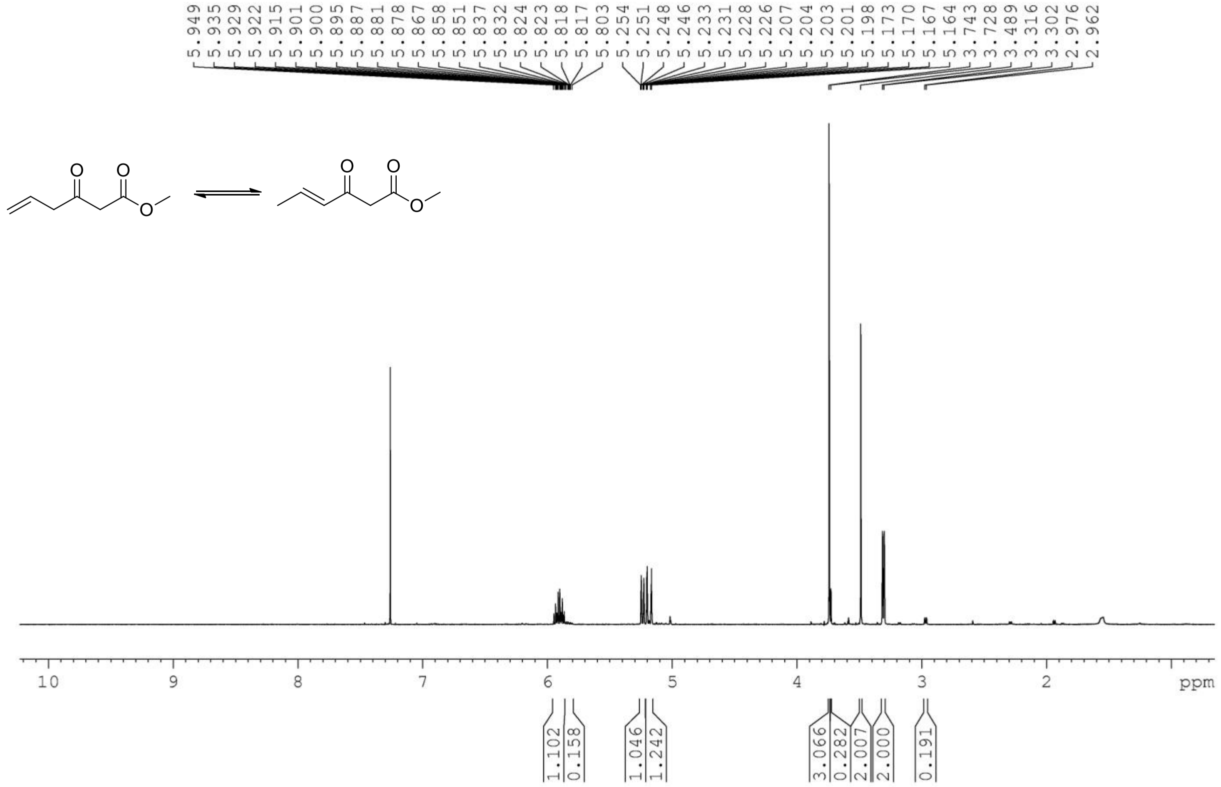

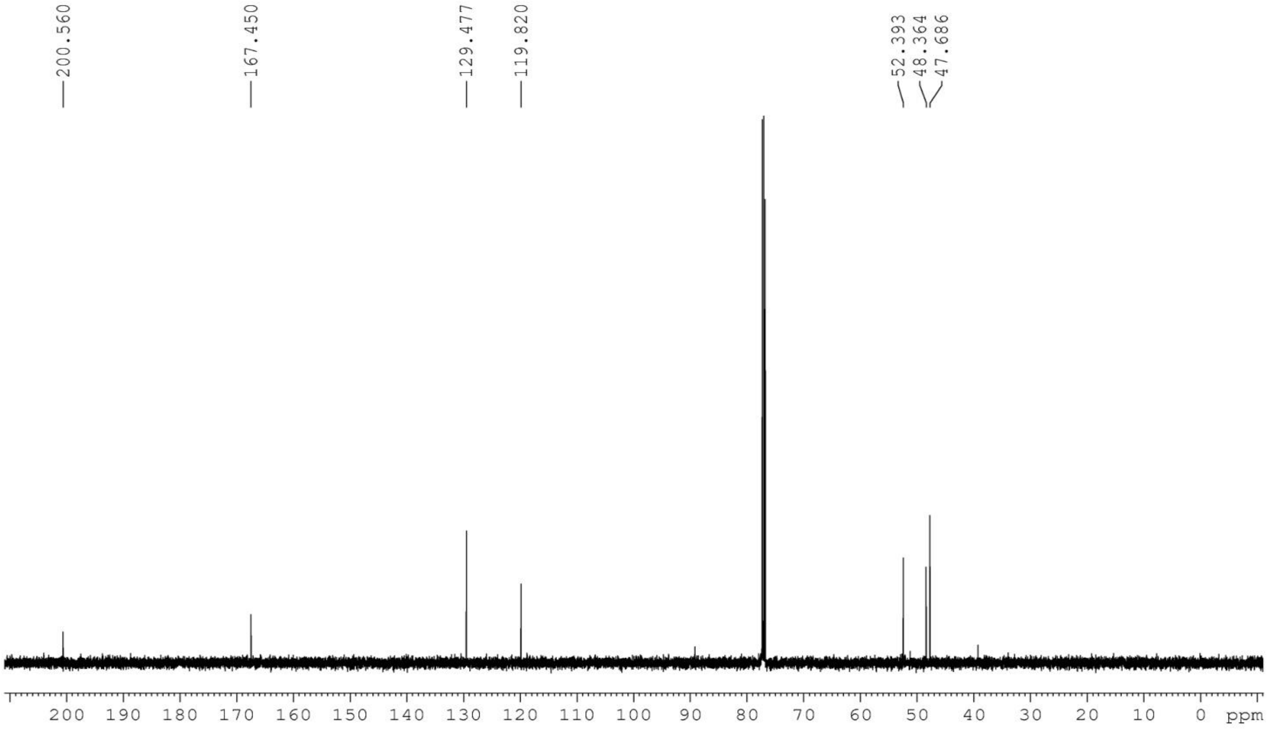

1.2 *Methyl 3-hydroxyhex-5-enoate*, **8** (CDCl$_3$)

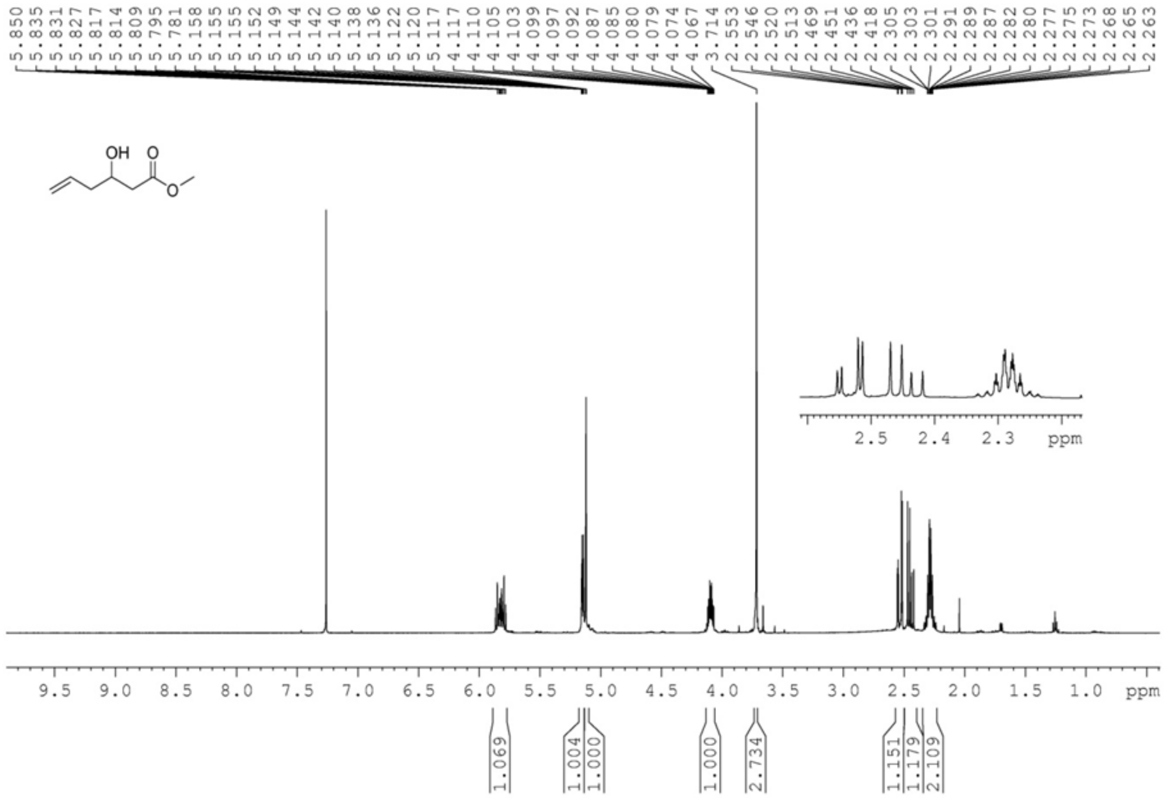

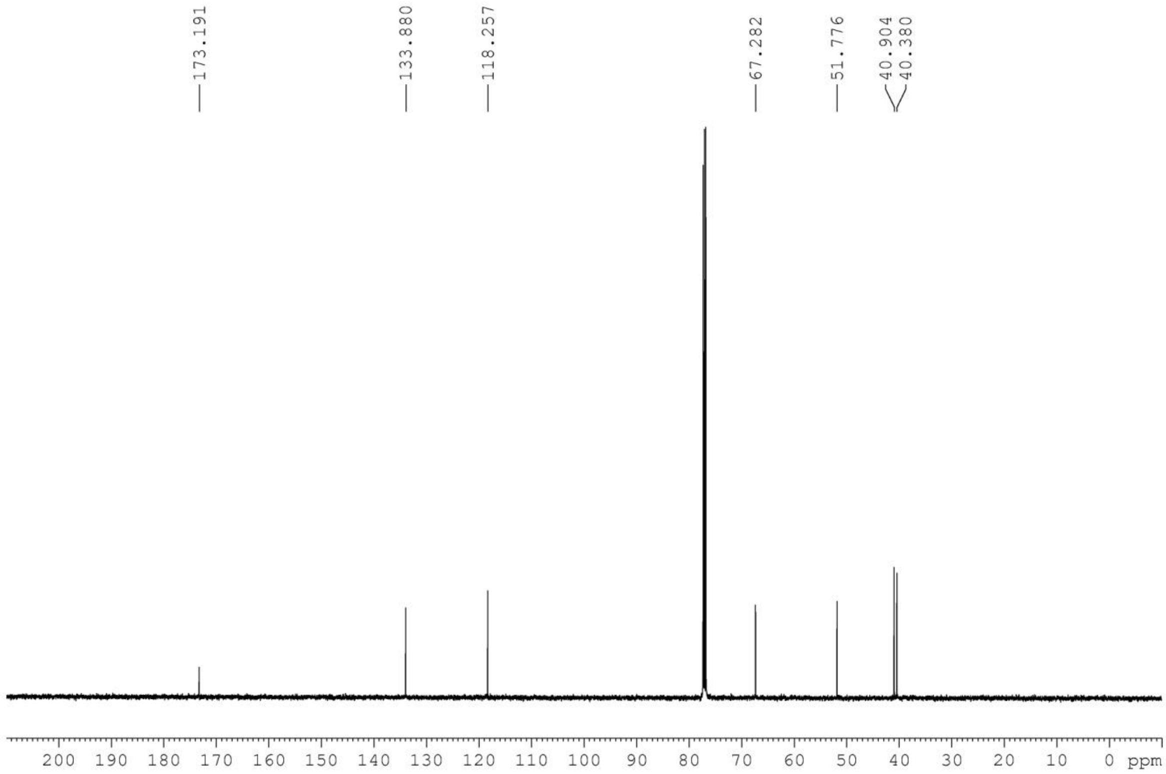

1.3 *tert-Butyl 3,5-dihydroxyoct-7-enoate*, **5** (CDCl$_3$)

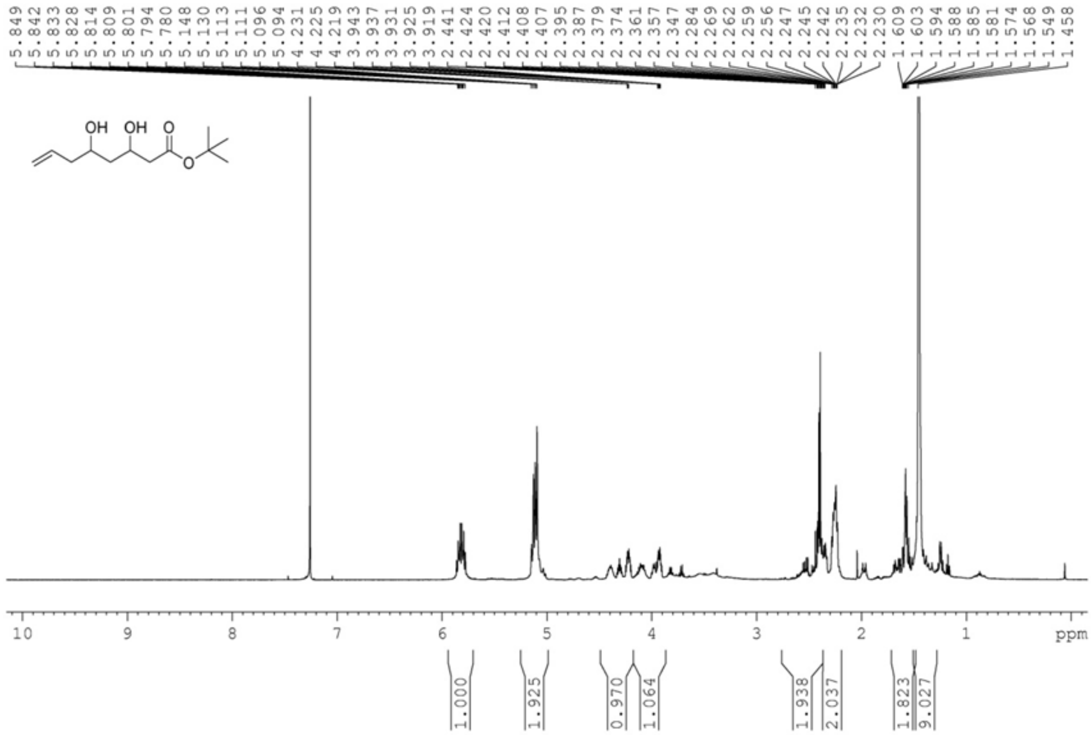

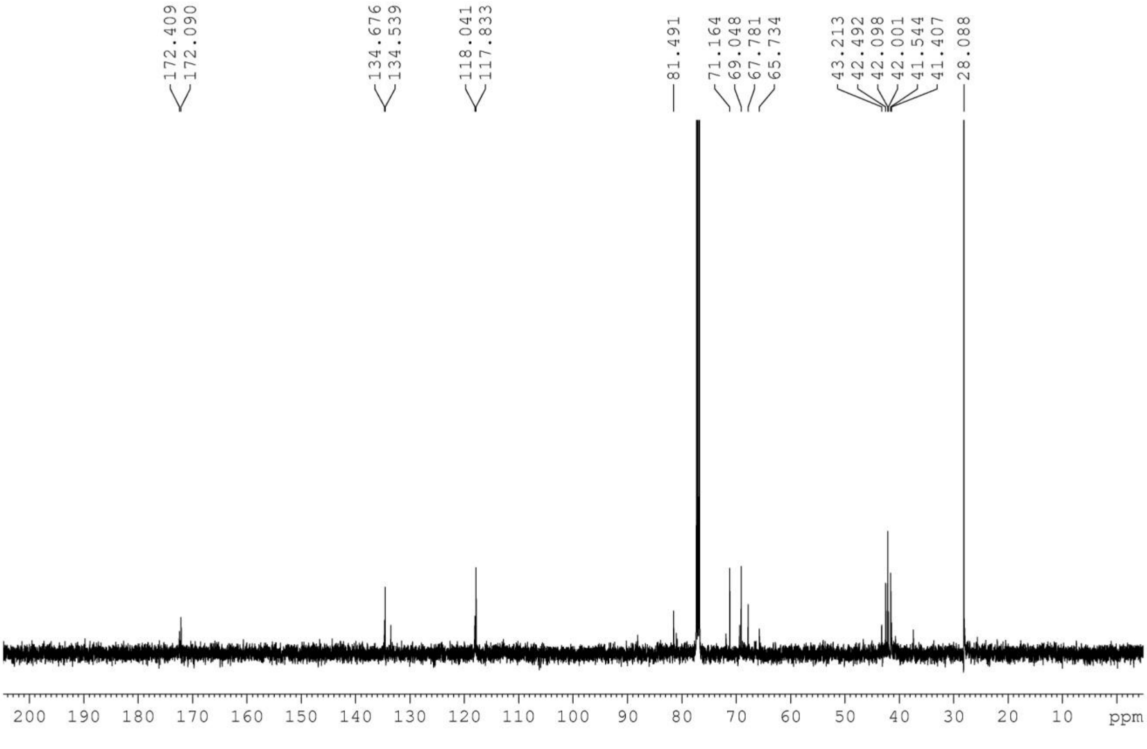

1.4 *Allyl-5,6-dihydro-2H-pyran-2-one*, **2** (CDCl$_3$)

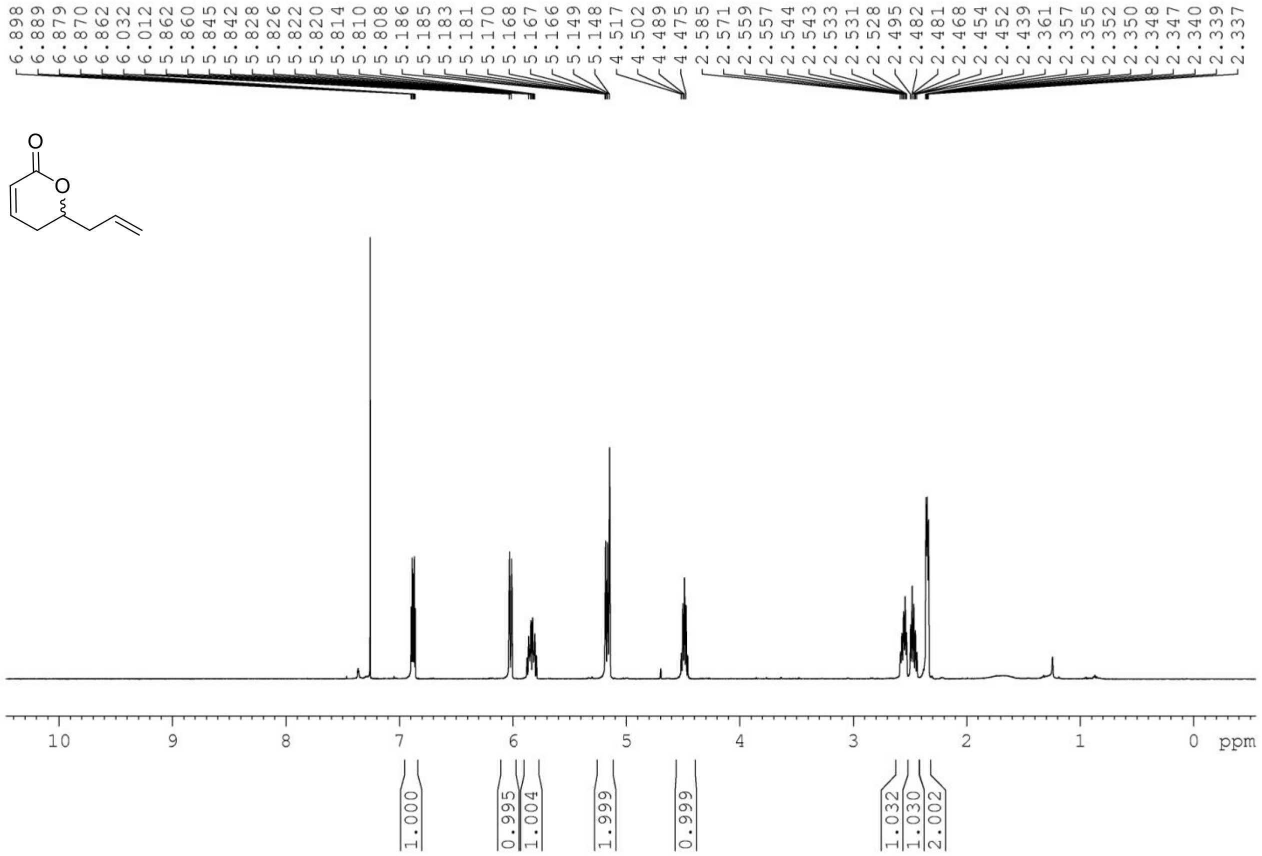

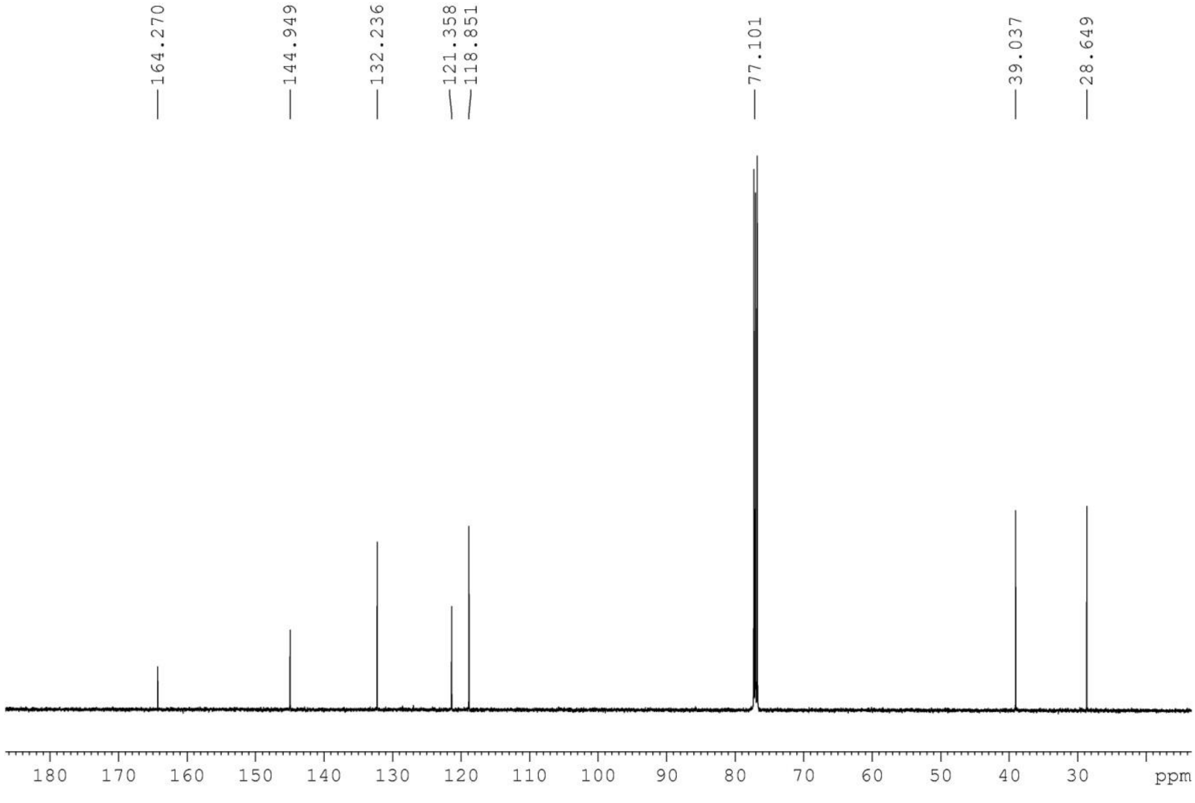

1.5 *5-phenylpent-1-en-3-ol*, **9** $(CDCl_3)$

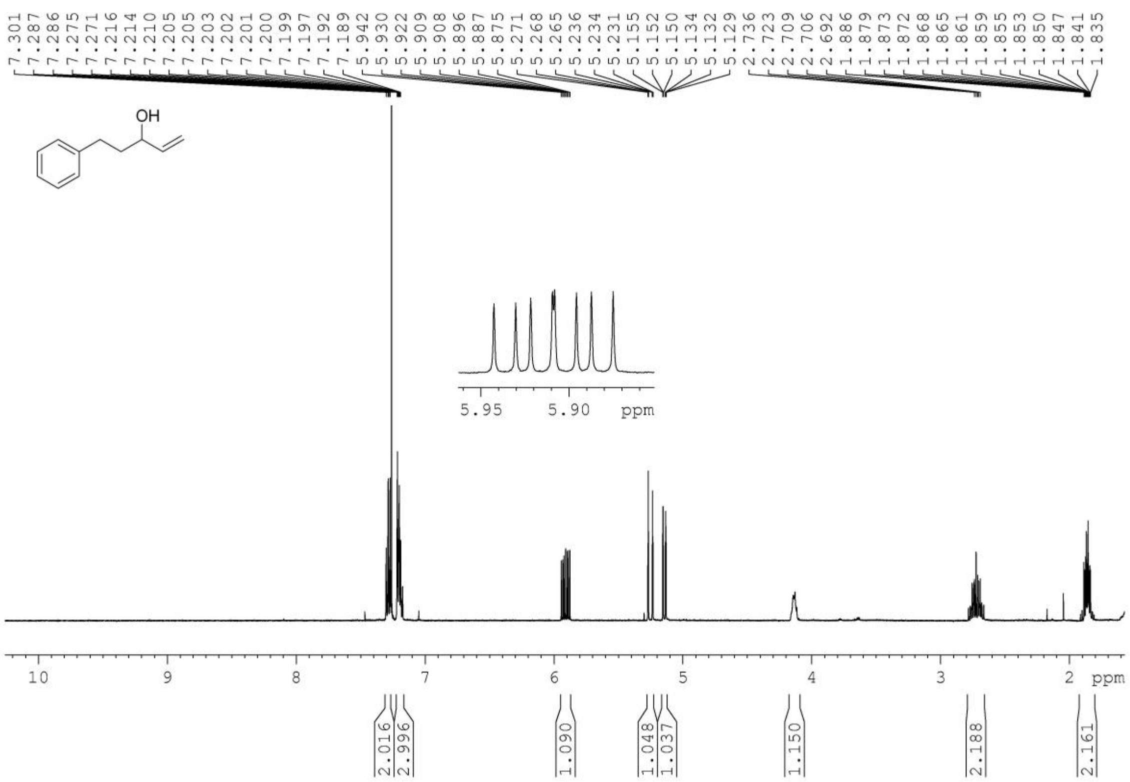

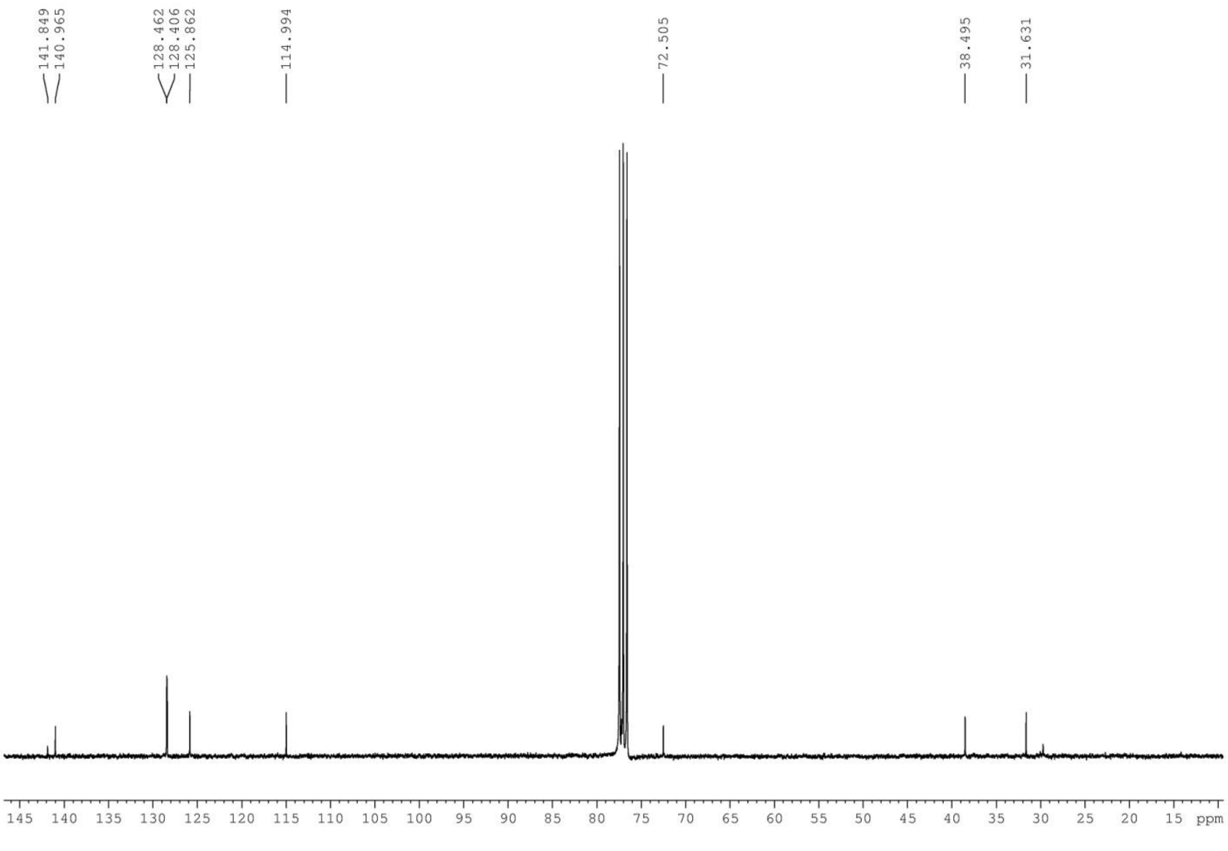

1.6 *5-phenylpent-1-en-3-one*, **3** ($CDCl_3$)

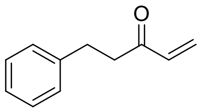

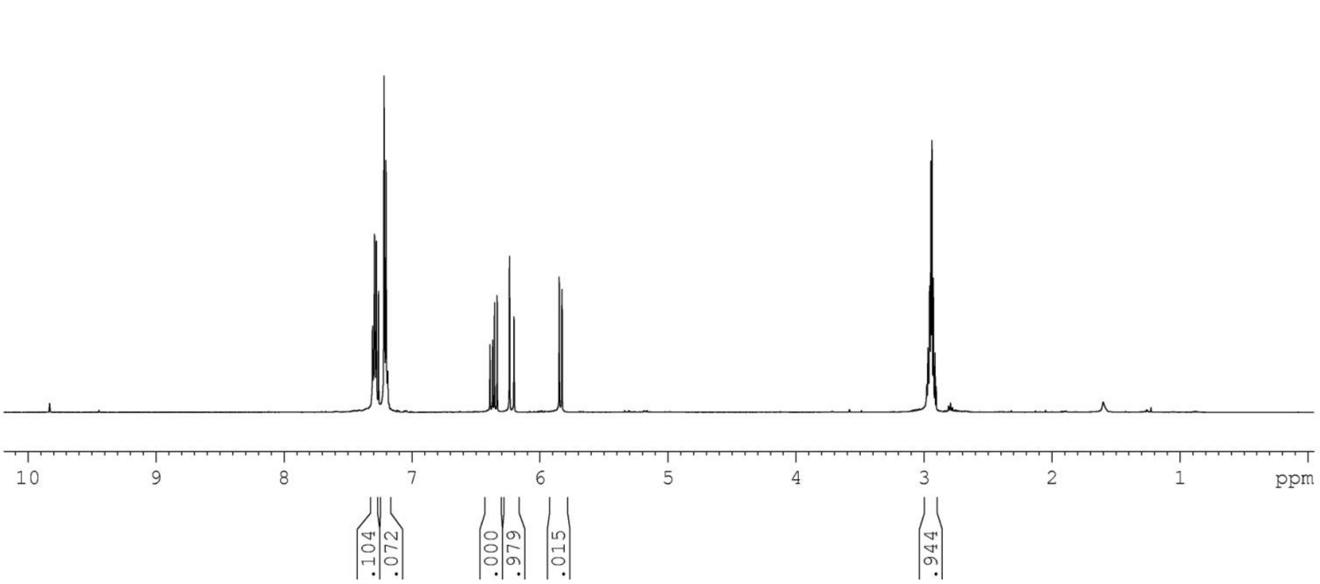

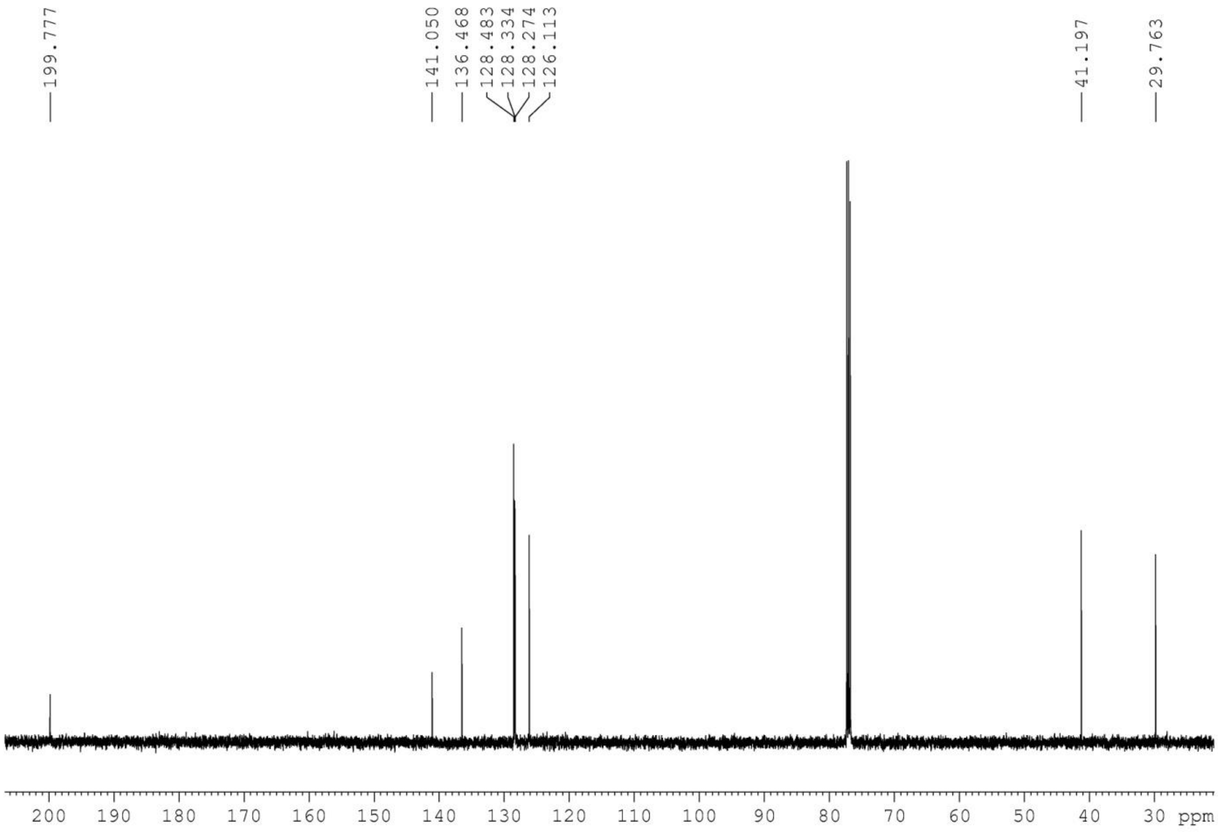

1.7 *(E)-6-(4-oxo-6-phenylhex-2-enyl)-5,6-dihydro-2H-pyran-2-one*, **1** ($CDCl_3$)

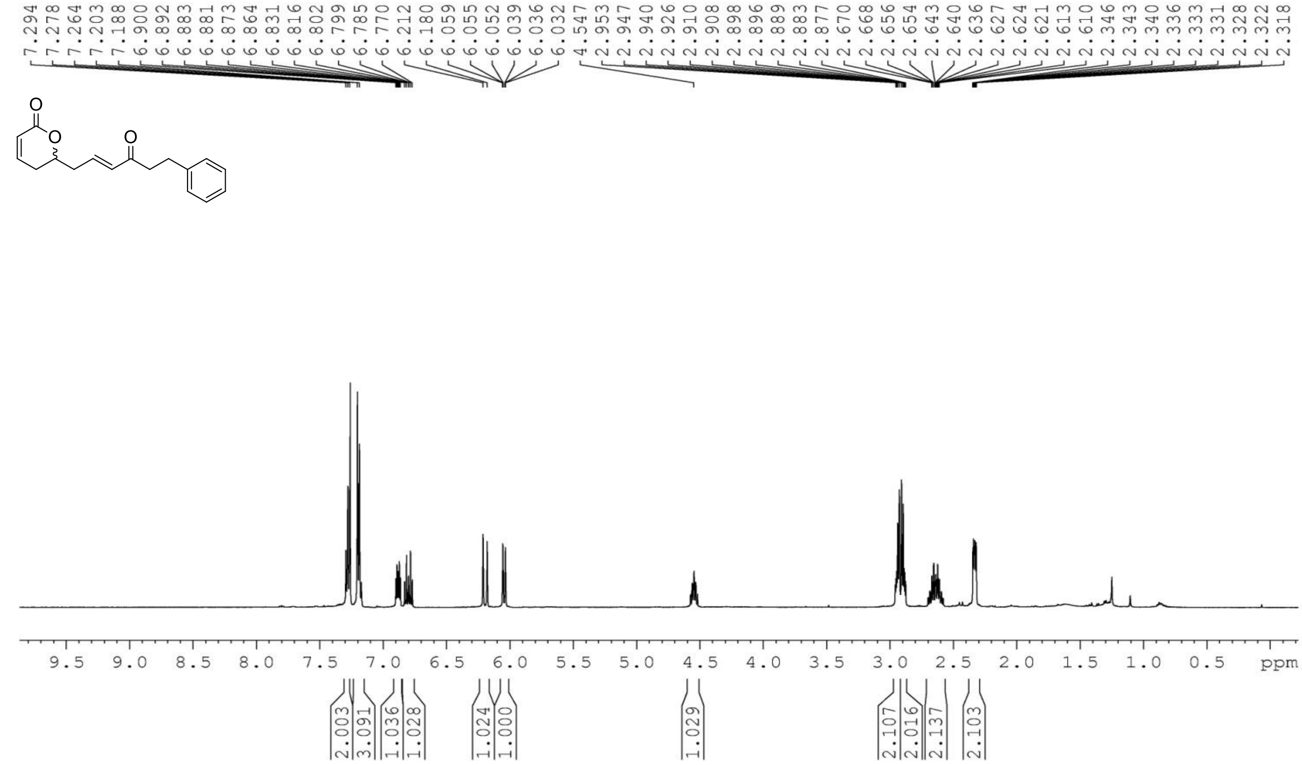

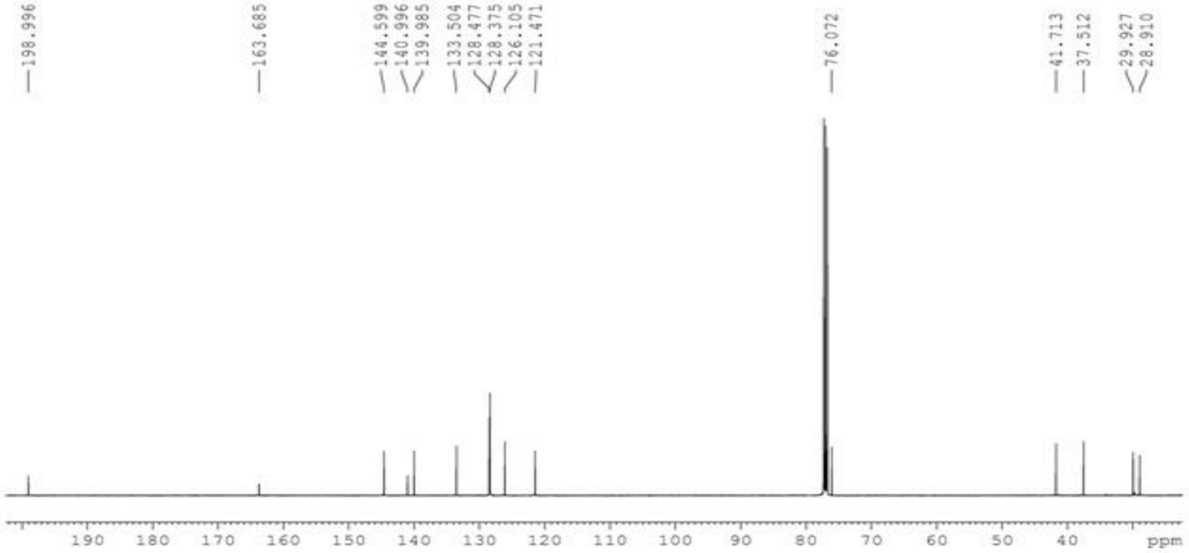

2. (*R*)-MPA ester of (*S*)-*methyl 3-hydroxyhex-5-enoate,* (*S*)-**8** (CDCl$_3$)

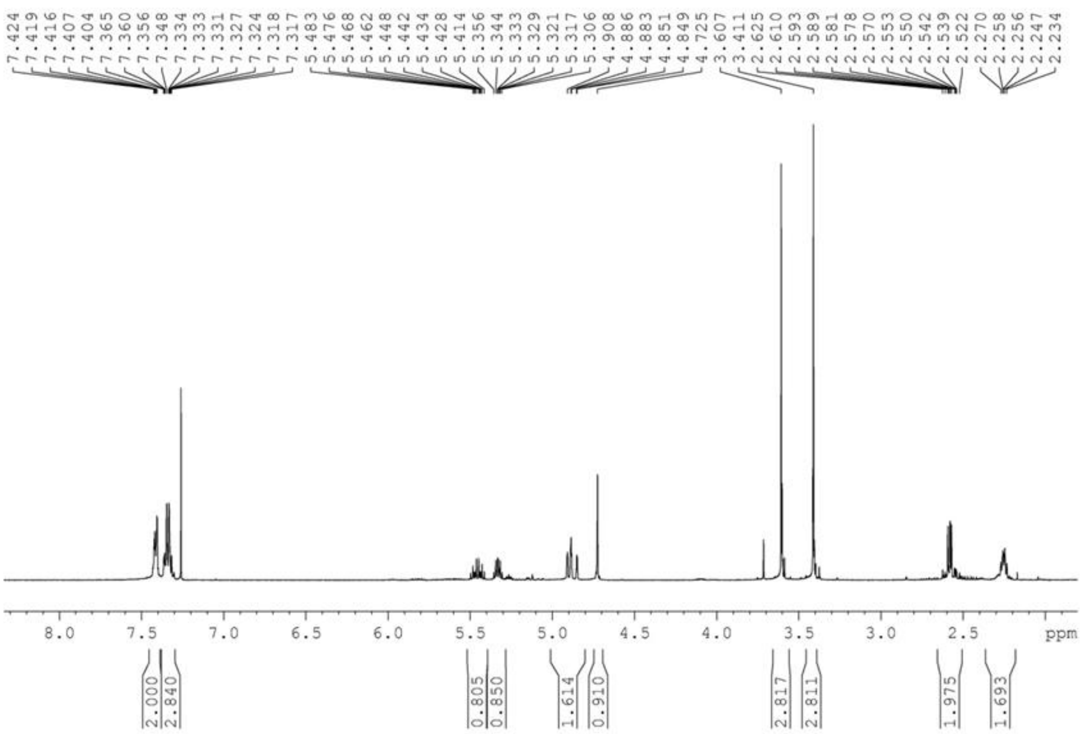

(*S*)-MPA ester of (*S*)-*methyl 3-hydroxyhex-5-enoate,* (*S*)-**8** (CDCl$_3$)

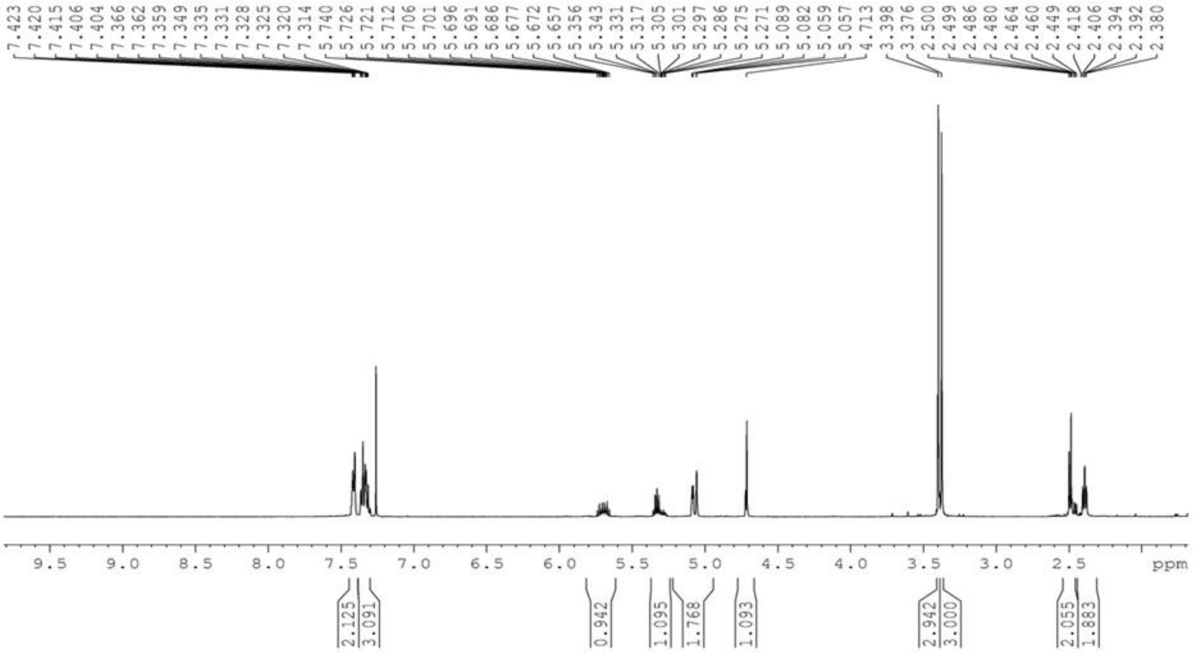

Supplement: Supplementary file 1 [file molecules-23-00640-s001.pdf]
